# HnRNP K mislocalisation is a novel protein pathology of frontotemporal lobar degeneration and ageing and leads to cryptic splicing

**DOI:** 10.1007/s00401-021-02340-0

**Published:** 2021-07-18

**Authors:** Alexander Bampton, Ariana Gatt, Jack Humphrey, Sara Cappelli, Dipanjan Bhattacharya, Sandrine Foti, Anna-Leigh Brown, Yasmine Asi, Yi Hua Low, Marco Foiani, Towfique Raj, Emanuele Buratti, Pietro Fratta, Tammaryn Lashley

**Affiliations:** 1 The Queen Square Brain Bank for Neurological Disorders, Department of Clinical and Movement Neuroscience, UCL Queen Square Institute of Neurology, London, UK; 2 Department of Neurodegenerative Diseases, UCL Queen Square Institute of Neurology, London, UK; 3 Icahn School of Medicine at Mount Sinai, New York, NY USA; 4 International Centre for Genetic Engineering and Biotechnology (ICGEB), Padriciano 99, 34149 Trieste, Italy; 5 The Firc Institute of Molecular Oncology Foundation (IFOM), Milan, Italy; 6 Duke-NUS Medical School, Singapore, Singapore; 7 University of Milan, Milan, Italy; 8 Department of Neuromuscular Diseases, UCL Queen Square Institute of Neurology, London, UK

**Keywords:** hnRNP K, Frontotemporal lobar degeneration, Frontotemporal dementia, RNA, Cryptic exons, Ageing

## Abstract

Heterogeneous nuclear ribonucleoproteins (HnRNPs) are a group of ubiquitously expressed RNA-binding proteins implicated in the regulation of all aspects of nucleic acid metabolism. HnRNP K is a member of this highly versatile hnRNP family. Pathological redistribution of hnRNP K to the cytoplasm has been linked to the pathogenesis of several malignancies but, until now, has been underexplored in the context of neurodegenerative disease. Here we show hnRNP K mislocalisation in pyramidal neurons of the frontal cortex to be a novel neuropathological feature that is associated with both frontotemporal lobar degeneration and ageing. HnRNP K mislocalisation is mutually exclusive to TDP-43 and tau pathological inclusions in neurons and was not observed to colocalise with mitochondrial, autophagosomal or stress granule markers. De-repression of cryptic exons in RNA targets following TDP-43 nuclear depletion is an emerging mechanism of potential neurotoxicity in frontotemporal lobar degeneration and the mechanistically overlapping disorder amyotrophic lateral sclerosis. We silenced hnRNP K in neuronal cells to identify the transcriptomic consequences of hnRNP K nuclear depletion. Intriguingly, by performing RNA-seq analysis we find that depletion of hnRNP K induces 101 novel cryptic exon events. We validated cryptic exon inclusion in an SH-SY5Y hnRNP K knockdown and in FTLD brain exhibiting hnRNP K nuclear depletion. We, therefore, present evidence for hnRNP K mislocalisation to be associated with FTLD and for this to induce widespread changes in splicing.

**Supplementary Information:**

The online version contains supplementary material available at 10.1007/s00401-021-02340-0.

## Introduction

Frontotemporal lobar degeneration (FTLD) describes a group of pathologically heterogeneous disorders all characterised by the selective and progressive atrophy of both frontal and temporal lobes [27, 66]. The ensuing neurocognitive syndrome broadly defined as frontotemporal dementia (FTD) is the second most common form of presenile dementia [8, 68]. FTD is itself an umbrella clinical term encompassing three distinct syndromes; behavioural variant FTD (bvFTD) and two language variants, progressive non-fluent aphasia (PNFA) and semantic dementia (SD) [12]. The current neuropathological classification of FTLDs recognises five major subgroups; three of which are characterised by specific proteinaceous inclusions principally containing TDP-43 (FTLD-TDP), FUS (FTLD-FUS) and Tau (FTLD-Tau) proteins [36, 38, 45, 53].

Two of these proteins, transactive response DNA-binding protein (TDP-43) and fused in sarcoma (FUS), are members of the diverse family of heterogeneous nuclear ribonucleoproteins (hnRNPs). HnRNPs are a group of ubiquitously expressed RNA-binding proteins (RBPs) implicated in the regulation of all aspects of nucleic acid metabolism including alternative splicing, mRNA stability, nucleo-cytoplasmic shuttling and translation [17]. They form highly dynamic co-operative complexes with RNA and other RBPs. The remodelling and restructuring of such complexes enables hnRNPs to bind and modulate an array of RNA regulatory machinery [23].

Disruption of RNA processing has been shown to be a central pathogenic mechanism in FTD and the overlapping motor disorder amyotrophic lateral sclerosis (ALS) [4]. Indeed, numerous transcriptomic and bioinformatic studies of TDP-43 and FUS depletion/mutation models have identified both gain and loss of splicing function phenotypes [7, 21, 29, 30, 49, 58, 67]. Recent studies have established that TDP-43 nuclear depletion induces the inclusion of normally repressed ‘cryptic exons’ into target transcripts [40, 48]. Cryptic exon inclusion can trigger premature polyadenylation leading to a sharp decline in protein expression, as in the case of *STMN2*/stathmin-2 [34, 48, 57]. Additionally, cryptic exon inclusion or constitutive exon skipping can trigger nonsense-mediated decay, or introduce evolutionarily untested maladaptive alterations to protein structure [30]. However, cryptic exon repression is not unique to TDP-43 and may indeed be an integral regulatory role of many RBPs to suppress the inclusion of intronic sequences by the spliceosome, as has been found for hnRNP C, RBM17, PTBP1, hnRNP L, SFPQ and MATR3 [2, 39, 47, 61, 69], but intriguingly, not for FUS [29, 30]. Understanding the transcriptional consequences of wider hnRNP dysfunction in the pathogenesis of FTLD is, therefore, imperative.

One of the most abundantly expressed proteins in the hnRNP family is hnRNP K. HnRNP K shares many structural characteristics typical of the hnRNP family including a nuclear localisation signal, a nuclear shuttling domain and three K homology (KH) domains which serve as nucleic acid recognition motifs [11, 50]. Unique to hnRNP K is its K interactive (KI) domain which facilitates its interaction with a multitude of protein targets within a vast interactome network [6, 55]. HnRNP K is widely expressed in multiple brain regions [63, 64] and has been specifically implicated in the post-transcriptional regulation of genes essential for maintaining ATP levels during cellular stress [22], axogenesis [41], myelination [37] and synaptic plasticity [19]. To date, research into the potential roles of aberrant hnRNP K expression in disease has been largely confined to cancer biology, where its overexpression and abnormal cytoplasmic accumulation have been correlated with advanced disease status and poor prognosis in several malignancies [5, 9, 10, 28].

Here we demonstrate, for the first time, hnRNP K mislocalisation from the nucleus to the cytoplasm as a frequent neuropathological feature in pyramidal neurons of the frontal cortex across the FTLD spectrum. We also present evidence for a degree of hnRNP K mislocalisation being a feature of the normal ageing process. We characterise this potentially novel pathological event with immunofluorescence to observe the spatial relationship of mislocalised hnRNP K puncta with the hallmark pathological inclusions of FTLD, markers of autophagy and mitochondria. We confirm hnRNP K mislocalisation to be a mutually exclusive pathology from TDP-43 or tau positive inclusions that are observed in different neuronal subtypes. We also utilise an hnRNP K knockdown cell model to predict the impact of hnRNP K depletion on the transcriptome. Analysis of cell-extracted RNA confirms the presence of cryptic exons specific to siRNA-induced nuclear loss of hnRNP K. Finally, we validate the presence of a cryptic exon inclusion event in postmortem human brain tissue, providing evidence for cryptic exon inclusion as a potentially direct functional consequence of hnRNP K nuclear depletion in FTLD neurons.

## Materials and methods

### Cases

Brains were donated to the Queen Square Brain Bank (QSBB) for neurological disorders (UCL Queen Square Institute of Neurology) and the Medical Research Council (MRC) Edinburgh Brain & Tissue Bank. All tissue samples were donated with the full informed consent. Accompanying clinical and demographic data of all cases used in this study were stored electronically in compliance with the 1998 data protection act and are summarised in Table 1. Ethical approval for the study was obtained from the NHS research ethics committee (NEC) and in accordance with the human tissue authority’s (HTA’s) code of practice and standards under licence number 12198.

**Table 1:** Cohort and clinical demographics

Case No	Clinical diagnosis	Pathological diagnosis	Age at disease onset	Age at death	Disease duration	Gender	Brain weight (g)	Mutations	Post mortem delay (h)
1*	AD	FTLD-TDP A	64	73	9	M	1252	C9orf72	61.1
2	CBD	FTLD-TDP A	51	61	10	M	1065		35.3
3	FTD	FTLD-TDP A	59	65	6	M	1176	C9orf72	30.0
4*	PNFA	FTLD-TDP A	66	72	6	M	1274		68.2
5	FTD	FTLD-TDP A	57	60	3	M	1673		40.4
6*	bvFTD	FTLD-TDP A	58	66	8	F	850	C9orf72	107.1
7	Control	FTLD-TDP A	75	79	4	M	–		10.0
8	FTD	FTLD-TDP A	52	58	6	M	1303	C9orf72	49.8
9	MND	FTLD-TDP A	47	53	6	M	1390		33.7
10*	FTD	FTLD-TDP A	53	63	10	M	955	C9orf72	77.3
11*	bvFTD	FTLD-TDP A	62	68	6	F	–	GRN (Q130fs)	99.8
12	MND	FTLD-TDP A	67	69	2	M	1398		62.5
13	CBD	FTLD-TDP A	75	79	4	F	1119		36.3
14*	PSP	FTLD-TDP A	83	87	4	F	1226		68.9
15*	FTD	FTLD-TDP A	57	62	5	M	–		92.9
16*	bvFTD	FTLD-TDP A	62	72	10	M	1320	TBK1	97.4
17	bvFTD	FTLD-TDP A	57	63	6	F	851		85.3
18	PNFA	FTLD-TDP A	57	62	5	F	981	C9orf72	63.1
19*	bvFTD	FTLD-TDP A	53	61	8	M	994	GRN (C31fs)	72.6
20*	FTD-MND	FTLD-TDP A	58	67	9	F	1000	GRN + C9orf72	115.0
21	FTD	FTLD-TDP A	62	68	6	M	1371	C9orf72	99.0
22*	bvFTD	FTLD-TDP A	43	45	2	M	1015	C9orf72	25.9
23	PNFA	FTLD-TDP A	56	67	11	F	789	C9orf72	85.6
24	FTD	FTLD-TDP A	59	71	12	F	1014	TBK1	76.0
25	bvFTD	FTLD-TDP A	49	55	6	M	974	GRN (C31fs)	29.3
26*	FTD-MND	FTLD-TDP A	66	74	8	F	782	C9orf72	85.8
27	FTD	FTLD-TDP A	54	60	6	M	1350	C9orf72	32.3
28	FTD-MND	FTLD-TDP A	66	71	5	M	1431	C9orf72	51.9
FTLD-TDP A Summary (*n* = 28)			59.6	66.1	6.5	18(M):10(F)	1142		64.0
29*	FTD-MND	FTLD-TDP B	63	67	4	F	1232		45.5
30	MND	FTLD-TDP B	67	69.0	2	M	1300		70.2
31	FTD	FTLD-TDP B	63	83	20	F	970		45.1
FTLD-TDP B Summary (*n* = 3)			64.3	73.0	8.7	1(M):2(F)	1167		53.6
32	FTD	FTLD-TDP C	52	65	13	F	899		27.7
33	bvFTD	FTLD-TDP C	64	66	2	F	1186	C9orf72	94.1
34	SD	FTLD-TDP C	73	83	10	M	1167		59.8
35	SD	FTLD-TDP C	58	72	14	F	972		31.2
36*	SD	FTLD-TDP C	67	76	9	M	1086		39.5
37	SD	FTLD-TDP C	58	73	15	F	976		37.9
38	SD	FTLD-TDP C	59	73	14	F	936		83.7
39	SD	FTLD-TDP C	64	78	14	M	1110		26.8
40	SD	FTLD-TDP C	64	74	10	M	1230		19.0
41	SD	FTLD-TDP C	50	65	15	M	1057		51.8
42	FTD	FTLD-TDP C	61	66	5	M	1117		70.8
43	PNFA	FTLD-TDP C	77	80	3	F	1502		26.3
FTLD-TDP C Summary (*n* = 12)			62.3	72.6	10.3	6(M):6(F)	1103		47.4
44	IBMPFD	FTLD-TDP D	–	48	–	F	1210	VCP	53.5
45	IBMPFD	FTLD-TDP D	53	71	18	M	1363		54.1
FTLD-TDP D Summary (*n* = 2)			53.0	59.5	18.0	1(M):1(F)	1287		53.8
46	bvFTD	FTLD-Tau	55	66	11	M	1208	MAPT (R406W)	60.8
47	PSP	FTLD-Tau	54	58	4	M	1225		78.3
48	bvFTD	FTLD-Tau	59	66	7	M	1399	MAPT (10 + 16)	58.2
49	bvFTD	FTLD-Tau	68	74	6	M	1048	MAPT (K280del)	125.0
50	bvFTD	FTLD-Tau	45	51	6	M	1046	MAPT (10 + 16)	52.6
FTLD-Tau Summary (*n* = 5)			56.2	63.0	6.8	5(M):0(F)	1185		75.0
51	ALS	FTLD-FUS	44	46	2	M	1570		96.0
52	ALS	FTLD-FUS	69	72	3	F	1268		93.0
FTLD-FUS Summary (*n* = 2)			56.5	59.0	2.5	1(M):1(F)	1419		94.5
53*	CBD	FTLD-Ni	48	54	6	F	1106		106.3
54	bvFTD	FTLD-Ni	50	57	7	M	1444		44.0
FTLD-Ni Summary (*n* = 2)			49.0	55.5	6.5	1(M):1(F)	1275		75.2
55	ALS	ALS	55	59	4	M	875		36.0
56	ALS	ALS	63	66	3	F	1316		66.0
57	ALS	ALS	58	63	5	F	1228	C9orf72	66.5
58	ALS	ALS	40	53	13	F	1226		42.3
59	ALS	ALS	77	80	3	F	1086		5.0
60	ALS	ALS	80	84	4	M	1453		56.3
61	ALS	ALS	74	76	2	M	1138	C9orf72	26.4
ALS Summary (*n* = 7)			63.9	68.7	4.9	3(M):4(F)	1189		42.6
62*	Control	Control	–	69	–	M	1435		171.0
63	Control	Control	–	67	–	M	1350		2.5
64	Control	Control	–	73	–	F	1214		24.0
65*	Control	Control	–	88	–	M	1077		16.3
66*	Control	Control	–	79	–	F	1288		88.8
67*	Control	Control	–	86	–	F	1234		120.0
68	Control	Control	–	93	–	F	1128		29.7
69	Control	Control	–	83	–	F	1263		99.0
70*	Control	Control	–	68	–	F	1330		45.1
71	Control	Control	–	70	–	M	1544		53.5
72	Control	Control	–	92	–	M	1213		46.3
73	Control	Control	–	96	–	F	1032		60.0
74	Control	Control	–	91	–	F	1130		71.8
75	Control	Control	–	84	–	F	–		40.6
76	Control	Control	–	80	–	M	–		11.5
77*	MSA	Control	–	83	–	M	1244		105.5
78	Control	Control	–	94	–	F	–		27.0
79	Control	Control	–	29	–	M	1590		44.0
80	Control	Control	–	25	–	M	1640		53.0
81	Control	Control	–	30	–	M	1670		71.0
82	Control	Control	–	25	–	M	1500		81.0
83	Control	Control	–	28	–	M	1330		38.0
84	Control	Control	–	34	–	M	1530		99.0
85	Control	Control	–	39	–	M	1360		76.0
86	Control	Control	–	37	–	F	1360		126.0
87	Control	Control	–	39	–	M	1470		86.0
88	Control	Control	–	46	–	M	1380		76.0
89	Control	Control	–	46	–	F	1400		99.0
90	Control	Control	–	48	–	M	1480		58.0
91	Control	Control	–	50	–	M	1350		122.0
92	Control	Control	–	57	–	M	1600		70.0
93	Control	Control	–	58	–	M	1650		96.0
94	Control	Control	–	53	–	F	1420		107.0
95	Control	Control	–	57	–	F	1320		73.0
96	Control	Control	–	51	–	M	1460		52.0
Control Summary (*n* = 35)			–	61.4	–	21(M):14(F)	1375		69.7

*RNA extracted from frozen frontal cortex tissue for cryptic exon validation*;* AD Alzheimer's disease; *aFTLD-U* atypical FTLD with ubiquitin inclusions; *ALS* Amyotrophic lateral sclerosis; *CBD* Corticobasal degeneration; *FTD* Frontotemporal dementia; *FTLD* Frontotemporal lobar degeneration; *FTLD-Ni* FTLD with no (known) inclusions; *bvFTD* Behavioural variant FTD; *IBMPFD* Inclusion body myopathy with early onset Paget disease; *PNFA* Progressive non-fluent primary aphasia variant FTD; *LPA* Logopenic aphasia; *MSA* Multiple system atrophy; *NIFID* Neuronal intermediate filament inclusion disease; *PPA* Primary progressive aphasia; *PSP* Progressive supranuclear palsy; *SD* Semantic dementia variant FTD; *SVD* Small vessel disease; Mutations: *C9orf72* Chromosome 9 open reading frame 72, *GRN* Progranulin, *MAPT* microtubule-associated protein tau, *TBK1* TANK-binding kinase 1, *VCP* Valosin-containing protein

All cases were diagnosed pathologically according to current consensus criteria [35, 45]. The cohort included pathologically diagnosed cases of FTLD-TDP A (*n* = 28), FTLD-TDP B (*n* = 3), FTLD-TDP C (*n* = 12), FTLD-TDP D (*n* = 2), FTLD-Tau (*n* = 5), FTLD-ni (*n* = 2), FTLD-FUS (*n* = 2), ALS (*n* = 7) and neurologically normal controls (*n* = 35).

### Immunohistochemistry

Slides with 8 µm mounted tissue sections were incubated at 60 °C overnight. Sections were deparaffinised in Xylene and rehydrated in decreasing grades of alcohol. Slides were incubated in methanol/hydrogen peroxide (0.3%) solution for 10 min to block endogenous peroxidase activity. For heat-induced antigen retrieval, slides were then transferred to a boiling solution of 0.1 M citrate buffer (pH 6.0) and pressure cooked at maximum pressure for 10 min. Slides were then incubated in 10% non-fat milk for 30 min at room temperature to block non-specific binding. Sections were incubated with primary antibody for 1 h at room temperature. The antibodies used for IHC in this study were both mouse-derived monoclonal hnRNP K antibodies (Abcam ab23644, 1:1000 and Bio-Rad MCA2622, 1:100). After three 5-min washes in tris-buffered saline with tween (TBS-T); slides were incubated for 45 min in 200 µl of biotinylated goat anti-mouse IgG secondary antibody (Vector Laboratories BA 9200, 1:200). Slides were washed as before and then incubated in pre-conjugated Strept(avidin)–Biotin Complex (ABC; DAKO) for signal amplification. The slides were then washed for a final time before being submerged in 3,3ʹ-Diaminobenzidine (DAB) chromogen and then counterstained in Mayer’s haematoxylin (BDH). Finally, slides were dehydrated in increasing grades of alcohol (70, 90 and 100% IMS), cleared in xylene and mounted.

### Quantitative pathological assessment of hnRNP K localisation

HnRNP K-stained frontal and temporal tissue sections were scanned using a Olympus VS120 slide scanner at 20 × magnification. The region of interest (ROI) was digitally marked, cropped and extracted using the Olympus VS desktop software to minimise file size. Extractions were consistently performed in the grey matter region of the second frontal gyrus for frontal lobe sections. Extracted ROI images were launched in ImageJ (v1.41) and a macro was used to generate the maximum number of random, non-overlapping, 1000 × 1000 pixel of 0.345 mm^2^ sized sample images (< 300) from each ROI. Across the cohort, the mean number of images analysed was 129 or 44.5 mm^2^ of analysed tissue per ROI.

Neurons with normal hnRNP K staining, as defined by a predominantly nuclear localisation of the protein, were detected and quantified by a supervised machine learning algorithm. We implemented a MATLAB-based program to generate the training data sets, which consisted of 250 images of frontal cortex neurons from different brain samples. AlexNet convolutional neural network with Adam optimizer [33] was used for training of the data sets and utilised a region based convolutional neural network (R-CNN) for identification of normally stained neurons. A manual estimation of the algorithm’s accuracy found the detection rate to be consistently above 80% with minimal false-positives.

Neurons with abnormal hnRNP K pathology, as defined by nuclear clearance of hnRNP K and its subsequent mislocalisation to the cytoplasm, were counted manually within each randomly generated image, blinded to disease status. The total degree of hnRNP K mislocalisation in each case was given as the proportion (%) of all images with at least one abnormal neuron counted within it.

### Double-label immunofluorescence

For double immunofluorescence, tissue sections were dewaxed, pre-treated and blocked as before. Sections were then either simultaneously or sequentially co-stained with mouse-derived hnRNP K (Abcam ab23644, 1:1000) and a second primary antibody. HnRNP K staining was amplified by incubation with a biotinylated IgG secondary antibody (DAKO/Vector laboratories, 1:200) prior to a 30 min incubation with ABC at room temperature as previously described for IHC. Antibody binding was visualised using a TSA Cyanine 3 amplification kit (Perkin-Elmer) which was applied to sections for 20 min at room temperature. After TBS-T washing, sections were incubated with species-appropriate Alexa Fluor 568 secondary antibodies (Invitrogen, 1:1000) for 2 h at room temperature to visualise the second (non-hnRNP K) antibody. Primary and secondary antibodies used for double immunofluorescence labelling as well as their respective conditions of incubation with anti-hnRNP K are detailed in Supplementary Table 1 (Online resource 1). Sections were washed a final three times in TBS-T with the second wash incorporating a 10-min incubation with 4ʹ,6-diamino-2-phenylindole (DAPI, Invitrogen, 1:1000) nuclei counterstain. Slides were mounted using Vectashield anti-fade mounting medium (Vector Laboratories).

Cross-reactivity was controlled for by the addition of two control sections stained as above with the individual omission of each primary antibody. Representative fluorescent images were captured at 20 × , 40 × or 63 × magnification using a Leica DM5500B fluorescence microscope and Z-stacks were subjected to a blind 3D deconvolution. Antibody staining was identified and imaged using the appropriate fluorescent channels, and colocalisation was confirmed or refuted on the combined, maximum-projected images.

### Cell culture

Human neuroblastoma SH-SY5Y cells (ATCC) (Invitrogen, ThermoFisher) were cultured in DMEM: F-12, 1:1 Mixture (SigmaAldrich) supplemented with 15% fetal bovine serum (Gibco-BRL, Life Technologies Inc.), 1% antibiotic–antimycotic-stabilised suspension (SigmaAldrich) and 1% MEM Non-essential Amino Acid solution without L-glutamine (SigmaAldrich) at 37 °C in an incubator with a humidified atmosphere of 5% CO_2_. SH-SY5Y cells were used up to 20 passages.

### Generation of SH-SY5Y hnRNP K knockdown line

SH-SY5Y cells (80 × 10^4^) were seeded in 6-well plates and transfected with a mix composed of 150 μl Opti-MEM (Life-Technologies), 3 μl of 40 μM siRNA and 9 μl Lipofectamine RNAiMAX (Invitrogen, ThermoFisher) for a final siRNA concentration of 80 nM. Gene knockdown was performed according to the manufacturer’s instructions. The siRNA sense sequences used for transfections were fire-fly luciferase (control) 5ʹ-uaaggcuaugaagagauac-3ʹ and (hnRNP K) 5ʹ-aauauuaaggcucuccguaca-3ʹ. After 48 h, cells were harvested for further analysis. Three replicates were performed for each condition.

### SH-SY5Y RNA extraction and sequencing

RNA was obtained using a miRNeasy kit (Qiagen), following the manufacturer’s instructions. RNA was recovered in 20–30 μl of RNase-free water and quantified with a Biophotometer D30 (Eppendorf, Hamburg, Germany). 9 μl (concentration from 5 to 9 μg) were sent to Novogene for library preparation and sequencing using their NEB unstranded protocol.

Briefly, mRNA was purified from total RNA using poly-T oligo-attached magnetic beads and then fragmented randomly by addition of a fragmentation buffer. First strand cDNA was synthesized using random hexamer primer and M-MuLV Reverse Transcriptase (RNase H-). Second strand cDNA synthesis was subsequently performed using DNA Polymerase I and RNase H. Double-stranded cDNA was purified using AMPure XP beads. Remaining overhangs of the purified double-stranded cDNA were converted into blunt ends via exonuclease/polymerase activities. After adenylation of 3' ends of DNA fragments, NEBNext Adaptor with hairpin loop structure was ligated to prepare for hybridization. To select cDNA fragments of preferentially 150 ~ 200 bp in length, the library fragments were purified with the AMPure XP system (Beckman Coulter, Beverly, USA). Finally, the final library was prepared by PCR amplification and purification of PCR products by AMPure XP beads. Paired-end 150 bp reads were sequenced on an Illumina NovaSeq and base-called with CASAVA.

Each sample was aligned to the human genome (build hg38) using STAR v2.7.2 [16] using GENCODE v30 [20] as the transcript reference. Unique alignment rates for each sample were all above 90%. Gene abundance was estimated using RSEM v1.3.1 [42] using the GENCODE v30 transcript set. Differential expression was assessed by inputting the RSEM transcript counts summed for each gene and running DESeq2 [44] with apeglm fold-change shrinkage [70]. Genes were considered differentially expressed at a FDR < 0.05 and a |log2FoldChange|> 1, although we note that hnRNP K itself fell just short of these cutoffs (log2 fold change = − 0.91; adjusted *p* value = 1.3e-7). Full differential expression results are available in Supplementary Table 2 (Online resource 1).

### SH-SY5Y western blotting analysis

Total protein extracts were obtained by sonicating cells for 10 min and homogenising them at high power with a BioRuptor UCD-200 in a mild lysis buffer composed of 1 × Phosphate Saline Buffer (PBS) and 1 × Complete Protease Inhibitor Cocktail (Roche Diagnostics). Protein extract (10 μg) from each sample was resuspended in 1 × NuPage LDS Sample Buffer (Invitrogen) prepared with 2.5% beta-mercaptoethanol and boiled at 95 °C for 5 min. Lysates were resolved on NuPage 10% Bis–Tris gel (Invitrogen) and transferred to a Nitrocellulose Power Blotter Select Transfer Stack (Invitrogen). After blocking with 4% milk, blots were probed with antibodies: polyclonal rabbit a-hnRNP K 1:250 (SigmaAldrich) and polyclonal mouse a-GAPDH 1:1000 (Santa Cruz Biotechnology). After washing, blots were probed with HRP-conjugated secondary antibody (Dako) and developed with SuperSignal West Femto (ThermoFisher) or ECL Luminata Classico Western HRP substrate (Merck Millipore). Images were acquired and analysed using Alliance 9.7 Western Blot Imaging System (UVItec Limited). Statistical analysis of protein expression was carried out by an unpaired *t* test on three independent experiments.

### Identification of cryptic exon targets from RNA-seq data

Differential splicing was assessed using Leafcutter, a tool optimised for finding novel splicing by clustering overlapping splice junction reads and comparing the contribution of each junction between conditions [43]. Briefly, splice junction reads were extracted from each alignment file using Regtools [18]. Junctions were clustered together with the following parameters: minimum proportional contribution to a cluster = 0.001, minimum read contribution to a cluster = 60, maximum intron length = 200,000 bp. Differential splicing was tested using a Dirichlet multinomial model comparing the proportions of each junction within a cluster between hnRNP K knockdown siRNA and scrambled siRNA control. After correcting for multiple testing using a Benjamini–Hochberg false discovery rate threshold of 5%, 8355 clusters were differentially spliced. We generated a custom script to identify novel cassette exons (see code availability), based on whether the cluster contained three splice junctions in the correct orientation, with two junctions flanking a central exon (inclusion junctions) and a third junction spanning the length of the cluster (skipping junction), and whether the inclusion and/or skipping junctions were annotated in GENCODE (v30). Any putative cassette exon larger than 250 bp was removed. 2290 differentially spliced clusters were classified as cassette exons. Percent spliced in (PSI) of each cassette exon in each sample was calculated by taking the average read count of the two inclusion junctions and dividing it by the read count of the skipping junction. Direction of effect or delta PSI (dPSI) was calculated by taking the difference between the average PSI in hnRNP K knockdown and the controls. 1039 cassette exons had a |PSI|> = 10%. We then classified exons as either cryptic or skiptic by comparing the mean PSI_control_ with the PSI_hnRNPK_. Cryptic exons were initially classified based on novelty in annotation, but as the comprehensiveness of transcript annotation improves, the number of novel exons will shrink. Cassette exons with a PSI < 10% in controls and a dPSI > 10% were considered *cryptic exons*. 101 exons met these criteria, of which 49 were not annotated by GENCODE. Cassette exons with a PSI > 90% in controls and a dPSI < − 10% were considered *skiptic exons*. Overlaps between differential expression and differential splicing were computed by matching gene names. To compare genes containing differentially spliced cassette exons to a null, a set of genes with similar expression levels was selected using the mean DESeq2 log10(baseMean) plus or minus 1 standard deviation.

Phylogenetic *p* values (PhyloP) conservation scores [56] between the human (hg38) and 29 other mammalian genomes (27 primates, dog, mouse, and armadillo), were downloaded from the UCSC table browser [31]. Mean PhyloP scores for each cassette exon were calculated using the UCSCtools bigWigSummary function [32]. Full results for the differential splicing analyses (all exons at FDR < 0.05, without filtering an effect size) are available in Supplementary Table 3 (Online resource 1). Whether an exon is labelled as cryptic or skiptic is listed in the “exon_set” column. The locations of these exons are listed in hg38 coordinates in the “exon_coords_hg38”, whereas the coordinates for the containing introns are listed in “intron_coords_hg38”.

DNA FASTA sequences were created for each cassette exon as well as the flanking 100 bp upstream and downstream, with respect to the transcript strand, using BSgenome.Hsapiens.UCSC. hg38. For the three sets, the sequences from the included events were directly compared to the skipped events (and vice versa) using the differential enrichment mode of MEME [3], setting possible motifs to be within four and eight nucleotides. The full command for each run was meme < target.FASTA > -dna -neg < background.FASTA > -objfun de -mod zoops -minw 4 -maxw 8 -wg 11 -ws 1 -markov_order 0 -maxiter 50 -distance 0.001 -brief 1000 -shuf 2 -csites 1000 -nmotifs 3 -seed 0 -wnsites 0.8.This identified motifs that are enriched in one set compared to the other. Each comparison was run in both directions, resulting in six different MEME runs.

As a comparison to the MEME motifs, we counted how often a particular nucleotide sequence occurred one or more times in a set of sequences. We compared the proportions of each group of sequences with a $\chi ^{2}$ test of equal proportions. We performed this for all possible DNA kmers with lengths 2–4. All *p* values were adjusted for multiple testing using the Bonferroni correction. We report enrichment as the proportion of included exon sequences/proportion of skipped exon sequences. Full results are in Supplementary Tables 4, 5 and 6 (Online resource 1).

### RNA extraction from human postmortem brain tissue

RNA was isolated from frontal cortex tissue of nine brains exhibiting normal hnRNP K mislocalisation (5 controls, 4 FTLD-TDPA) and 12 brains exhibiting mislocalised hnRNP K (8 FTLD-TDP A, 1 FTLD-TDP B, 1 FTLD-TDP C, 1 FTLD-ni and 1 control) as indicated in Table 1. 50 mg of flash-frozen tissue was homogenised in 700 µL of Qiazol (Qiagen) using a TissueRuptor II (Qiagen). Chloroform was added and RNA subsequently extracted following the spin-column protocol from the miRNeasy kit with DNAse digestion (Qiagen). RNA was eluted off the column in 50 µL of RNAse-free water. RNA quantity and quality were evaluated using a spectrophotometer.

### PCR validation of cryptic exon targets

cDNA was synthesised from SH-SY5Y cell-derived and postmortem tissue-derived RNA using SuperScript IV VILO Master Mix with ezDNase enzyme step (ThermoFisher). Cryptic exons in predicted targets of hnRNP K were amplified using primer pairs that flank the cryptic exon, as well as a third primer which spans the cryptic exon. Primer sequences are presented in Supplementary Table 7 (Online resource 1). PCR for splicing events was conducted using 2 × GoTaq PCR Master Mix (Promega) using the following touchdown thermal cycling conditions: 95 °C for 5 min, (95 °C for 30 s, 75 °C for 45 s (− 1 °C per cycle), 72 °C for 1 min) × 15 cycles, (95 °C for 30 s, 60 °C for 45 s, 72 °C for 1 min) × 20 cycles, 72 °C for 5 min. Products were electrophoresed and visualised on agarose gels with SybrSafe (ThermoFisher) or using D1000 ScreenTape and reagents (Agilent) on the 2200 Tapestation system (Agilent).

PCR products from the amplification of the *FAM160B2* cryptic event in human postmortem tissue-derived cDNA were gel excised and DNA extracted using the QIAquick Gel extraction Kit (Qiagen). Sanger sequencing was performed (Source Bioscience, Cambridge, UK) and products were aligned using SnapGene to validate that the amplified product corresponded to a cryptic splicing event.

### Statistical analysis

For pathology, all generated data plots and accompanying statistical analyses were conducted using Graphpad Prism software (v7.00 for Windows). Data sets were subjected to the D’Agostino–Pearson test for normal variance which in-turn guided the selection of further statistical tests for *t* test comparisons and clinical data correlation purposes. In all statistical comparisons, a corresponding *p* value of < 0.05 was considered statistically significant. The level of significance is demonstrated in figures as * for *p* < 0.05, ** for *p* < 0.01, *** for *p* < 0.001. Where appropriate, for all data-plots provide the corresponding statistical test, *n* value, *p* value and *r* value are detailed in the figure legend.

## Results

### HnRNP K is frequently mislocalised in the frontal cortex

**Fig. 1:**
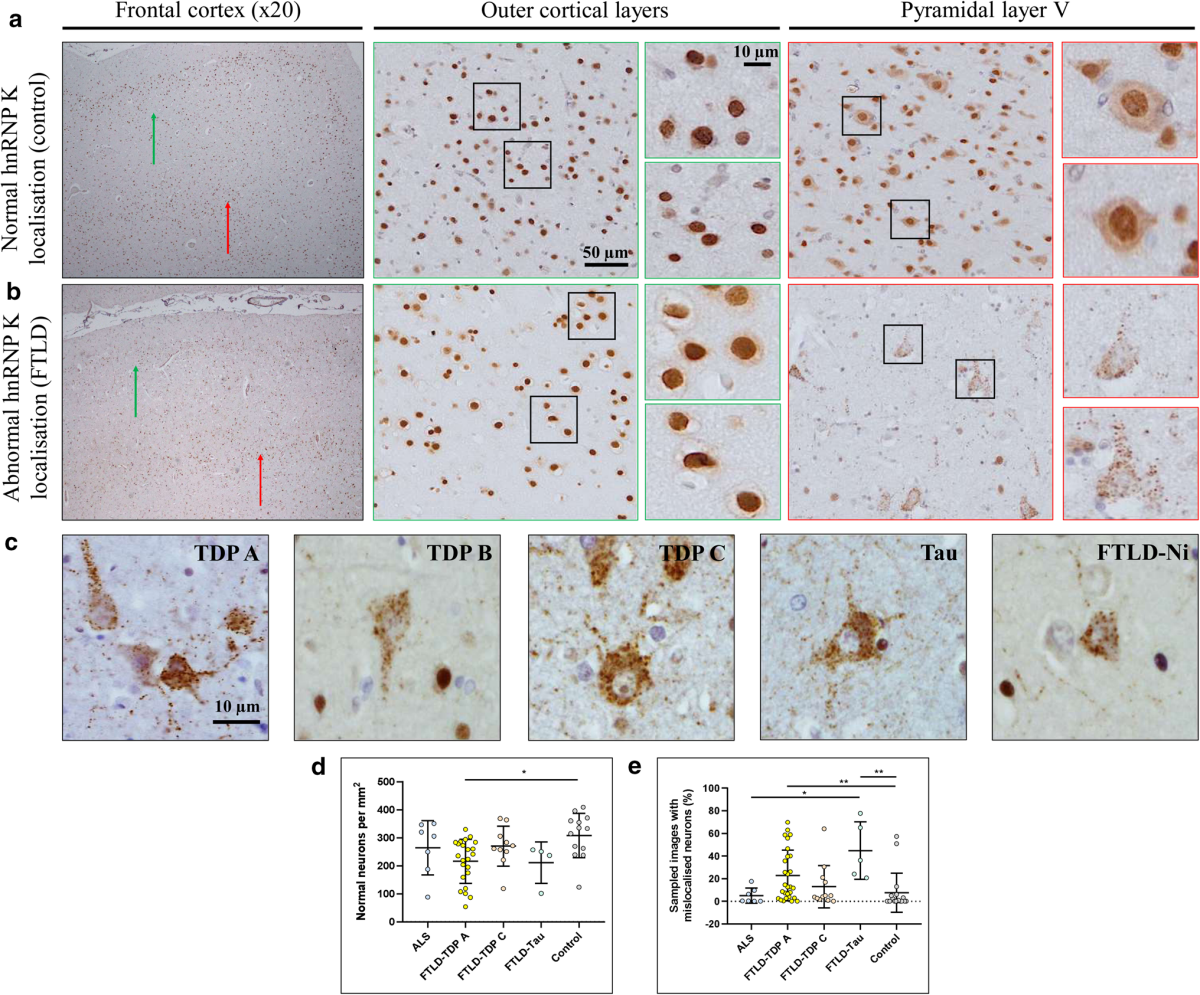
Immunohistochemical staining and quantitation of hnRNP K neuronal pathology in FTLD and control subjects. **a** Normal nuclear localisation of hnRNP K localisation within neurons of outer and pyramidal cortical layers. **b** FTLD-TDP A case with normal neuronal staining within outer cortical layers and abnormal staining within pyramidal neurons as defined by hnRNP K nuclear depletion and accumulation of cytoplasmic puncta. **c** Examples of hnRNP K mislocalisation in pyramidal neurons of FTLD subtypes (from left to right): TDP A, TDP B, TDP C, Tau and FTLD-Ni (no inclusions). **d** Age-matched controls (*n* = 18) showed significantly more normal hnRNP K-stained neurons per mm^2^ region analysed than the largest disease group, FTLD-TDP A (*n* = 28) (*p* = 0.013; Tukey’s test). **e** FTLD-TDP A and FTLD-Tau cases exhibited significantly more hnRNP K mislocalisation than controls expressed as proportion of sampled images with at least 1 (> 0) abnormal neuron (*p* = 0.004, *p* = 0.002). FTLD-Tau cases also exhibited significantly more mislocalised hnRNP K than ALS cases using both metrics of abnormality (*p* = 0.04)

Immunohistochemical staining of hnRNP K in the frontal cortex demonstrates normal neuronal localisation of the protein within the most superficial layers of the cortex (layers I–II). Neurons within these layers exhibited a predominantly nuclear localisation of the protein with uniform intensity irrespective of disease status (Fig. 1a, b). However, the larger pyramidal neurons of layers III and V revealed a remarkably different staining pattern in FTLD cases compared to age-matched controls, a potentially novel neuropathological event. In control brains, pyramidal neurons were characterised by a predominantly nuclear localisation of hnRNP K with only low-intensity perinuclear or cytoplasmic staining (Fig. 1a). By contrast, FTLD brains exhibited vast regions of severely abnormal hnRNP K mislocalisation that were occasionally observed across the entire pyramidal cell layers. Afflicted neurons were morphologically characterised by an almost complete depletion of nuclear hnRNP K and a concurrent punctate cytoplasmic accumulation of the protein that extended into the neurites (Fig. 1b).

### HnRNP K is mislocalised across frontotemporal lobar degeneration subtypes

Upon closer examination we identified many examples of this widespread mislocalisation across the FTLD-TDP and FTLD-Tau pathological spectrum and in a rare FTLD-ni subject with no known pathological inclusions (Fig. 1c, Supplementary Fig. 1a–d, Online resource 2). To quantify this novel neuropathological event, we sought to obtain large numbers of randomly generated images (average of 129 images per case or 44.5 mm^2^ of analysed tissue) that had been generated from immunohistochemically stained frontal cortex.

Age-matched cases of ALS, FTLD-TDP A, FTLD-TDP C, FTLD-Tau and control subjects were then selected for comparative analysis (Supplementary Fig. 1e, Online resource 2). Disease categories were compared according to their degree of normal hnRNP K staining by calculating each case’s average number of normally stained neurons per mm^2^ analysed. As expected, control cases had the greatest frequency of normal hnRNP K-stained neurons per image which was significant compared to the FTLD-TDP A disease subtype (*p* = 0.013) (Fig. 1d). This reflected control frontal cortex having a greater number of surviving neurons than FTLD subjects.

The same groups were then compared on their degree of abnormal hnRNP K staining by calculating the proportion of all sampled images which contained at least one mislocalised neuron for each case. For example, a case with a mislocalisation score of 50% would mean that 50% of all the case’s randomly sampled images contained at least one neuron with hnRNP K mislocalisation. By contrast to normal hnRNP K staining, FTLD-TDP A and FTLD-Tau disease groups exhibited significantly more mislocalisation of hnRNP K protein relative to controls (*p* = 0.004, *p* = 0.002) (Fig. 1e). There was no difference between controls and FTLD-TDP C subjects. Of interest, FTLD-Tau cases also exhibited greater hnRNP K mislocalisation than ALS subjects which were included as a disease control due to the relative sparing of the frontal cortex in ALS pathology (*p* = 0.04).

Notably, we did not find hnRNP K mislocalisation to be a specific pathological feature of any one FTLD subtype. Equally, not all individuals of any one subtype were vulnerable to hnRNP K mislocalisation. Indeed, the proportion of all neurons counted that were found to have mislocalised hnRNP K within the FTLD-TDP A group alone ranged from 0% to as much as 20% making this a very frequent neuropathological feature in some cases (Supplementary Fig. 1f, Online resource 2). This prompted us to explore the relationship of other variables with hnRNP K mislocalisation score and in particular age at death.

### HnRNP K mislocalisation is an age-related feature of neurodegenerative disease

**Fig. 2:**
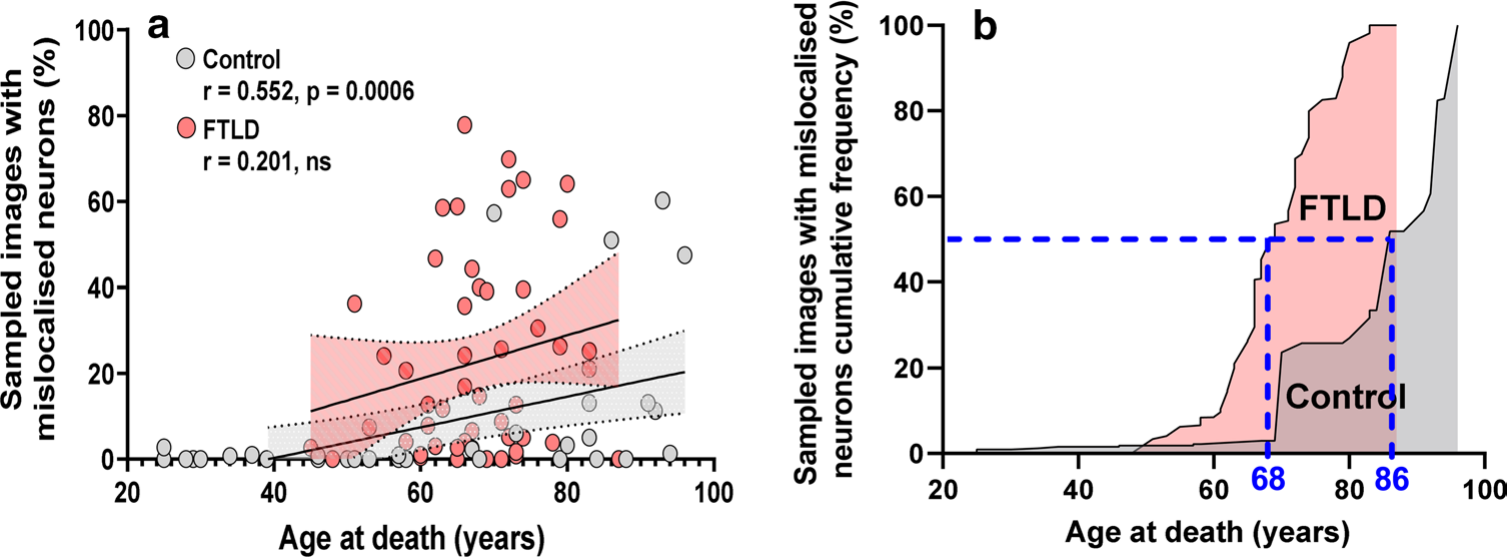
HnRNP K mislocalisation is an age-related pathology that is advanced in FTLD. **a** HnRNP K mislocalisation in controls (in grey, *n* = 35) was found to positively correlate with age at death (Spearman’s *r* = 0.552, *p* = 0.0006). HnRNP K mislocalisation in combined FTLD-TDP and FTLD-Tau subjects (in red, *n* = 50) was found to be only weakly associated with age at death (Spearman’s *r* = 0.201, ns). **b** Cumulative frequency plot of hnRNP K mislocalisation with ascending age normalised to total amount of quantified mislocalisation for each control/disease group. The intra-group median frequency of hnRNP K mislocalisation within the FTLD-TDP A group was 18 years in advance of the control cohort (dotted blue lines)

We analysed 35 control subjects with an age at death ranging over 70 years from 25 to 96 as well as a combined FTLD disease group (FTLD-TDP and FTLD-Tau subjects, *n* = 50) which involved pooling the previously analysed FTLD-TDP A, FTLD-TDP C and FTLD-Tau cohorts with the further addition of *n* = 3 and *n* = 2 FTLD-TDP B and FTLD-TDP D cases, respectively. Analysing control individuals in isolation, age at death was found to strongly correlate with hnRNP K mislocalisation (*r* = 0.552, *p* = 0.0006) (Fig. 2a). By contrast hnRNP K mislocalisation in the FTLD cohort was much more weakly associated with age at death (*r* = 0.201, *p* = 0.162 ns).

To better visualise and compare the different relationships between increasing age and hnRNP K pathology in control and FTLD groups, we generated a cumulative frequency plot (Fig. 2b). We plotted the rolling total of hnRNP K mislocalisation for each cohort with ascending age and normalised this to the sum total of each group’s total level of quantified mislocalisation. This illustrated the advanced nature of hnRNP K pathology onset in younger FTLD individuals relative to controls; with the median amount of mislocalisation in the FTLD’s (reached at 68 years of age) being 18 years earlier than in controls (86 years) (Fig. 2b).

### Mislocalised hnRNP K is distinct from TDP-43 and Tau inclusions

**Fig. 3:**
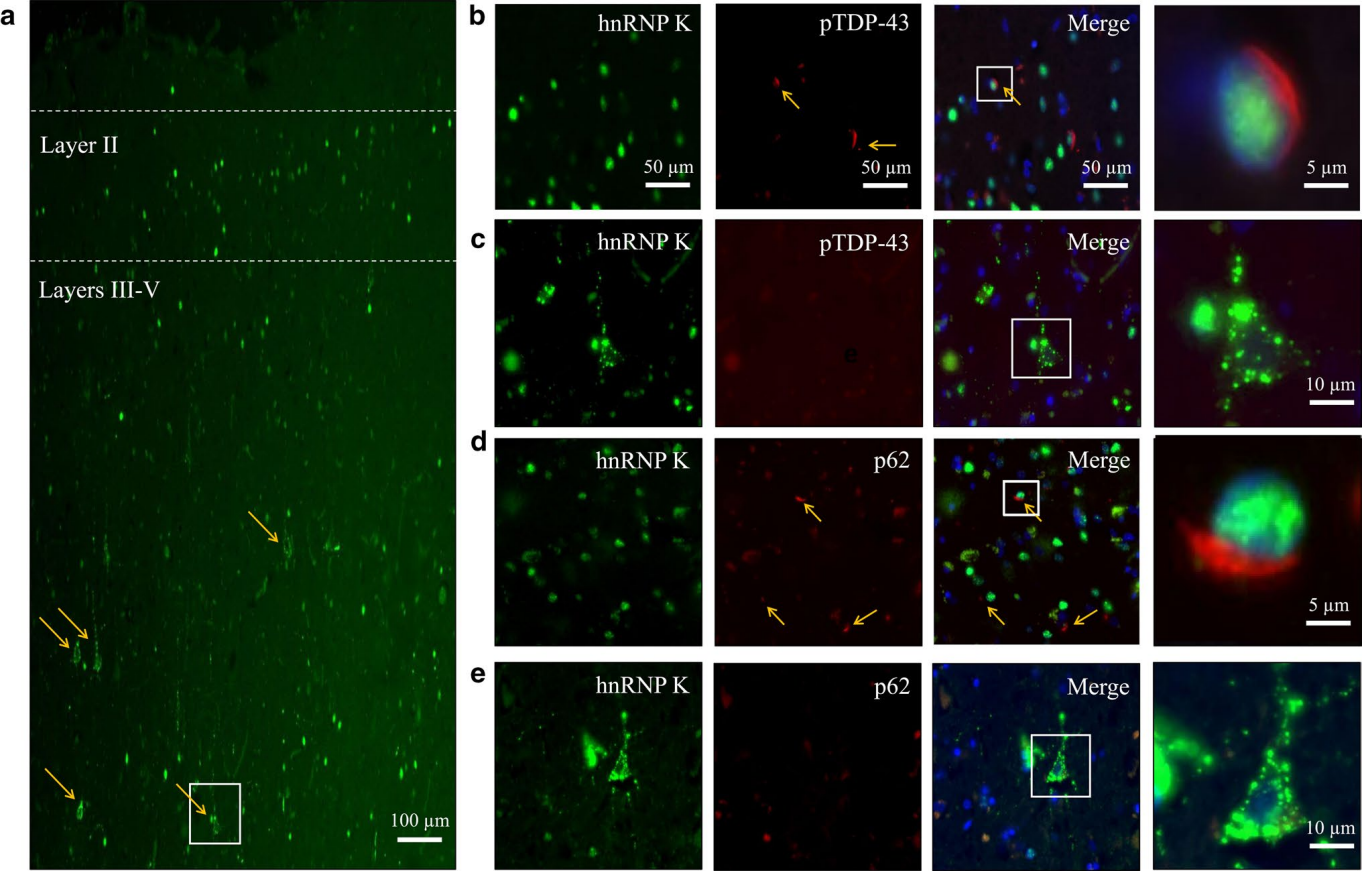
Neurons that exhibit hnRNP K mislocalisation are independent of those that contain TDP-43 inclusions and hnRNP K pathology is p62-negative. **a** HnRNP K immunofluorescence of frontal cortex (10 ×) in FTLD-TDP A showing the relative anatomical positioning of normal (layer II) and abnormal (pyramidal layers III & V) hnRNP K localisation. Arrows point to neurons with hnRNP K mislocalisation. **b** Representative images of double-label immunofluorescence in layer II neurons exhibiting normal hnRNP K staining with TDP-43 inclusions. **c** Representative images of pyramidal layer V neurons with mislocalised hnRNP K (as boxed in a) but no TDP-43 pathology, demonstrating that hnRNP K mislocalisation and TDP-43 pathologies do not co-occur in the same neurons. **d,** **e** Double-label immunofluorescence of hnRNP K and p62 in FTLD-TDP A cortical layers II and V showing neurons with p62-positive inclusions have normal nuclear localisation of hnRNP K (**d**) and that cytoplasmic puncta in pyramidal neurons with hnRNP K mislocalisation are p62-negative (**e**). Arrows point to TDP-43/p62-positive inclusions and scale bars are as indicated in the first row unless otherwise stated

To confirm that neurons with hnRNP K pathology were distinct from those with TDP-43 immunoreactive inclusions, we performed double fluorescence to determine the spatial relationship of these two pathologies in FTLD-TDP A. Neurons with TDP-43 inclusions that were predominantly found in cortical layer II displayed normal, nuclear-localised hnRNP K (Fig. 3a, b). However, we found that the cytoplasmic puncta of mislocalised hnRNP K in pyramidal neurons did not colocalise with cytoplasmic inclusions of TDP-43 in FTLD-TDP A (Fig. 3c). Indeed, a similar double-negative result was obtained with antibodies against the classical inclusion marker SQSTM1/p62 (Fig. 3d, e). Hence cytoplasmic hnRNP K puncta are unlikely to be components of ubiquinated inclusions.

Indeed, neurons with hnRNP K pathology were also found to be distinct from those with tau-positive inclusions within the frontal cortex of FTLD-Tau. Tau inclusions were readily identifiable within the pyramidal cell layers, but again, were not found within neurons exhibiting hnRNP K mislocalisation (Supplementary Fig. 2a, b, Online resource 2). In further attempts to characterise the subcellular location of hnRNP K cytoplasmic puncta in mislocalised neurons, double fluorescence was also performed with mitochondrial marker voltage-dependent anion channel (VDAC), classical autophagosome marker LC3 and stress granule/RNA-binding protein GTPase-activating protein-binding protein 2 (G3PB2). VDAC, LC3 and G3BP2 staining was principally cytoplasmic but no marker was enriched at the site of hnRNP K puncta (Supplementary Fig. 3a–f, Online resource 2).

### HnRNP K knockdown leads to widespread changes in gene expression and splicing

**Fig. 4:**
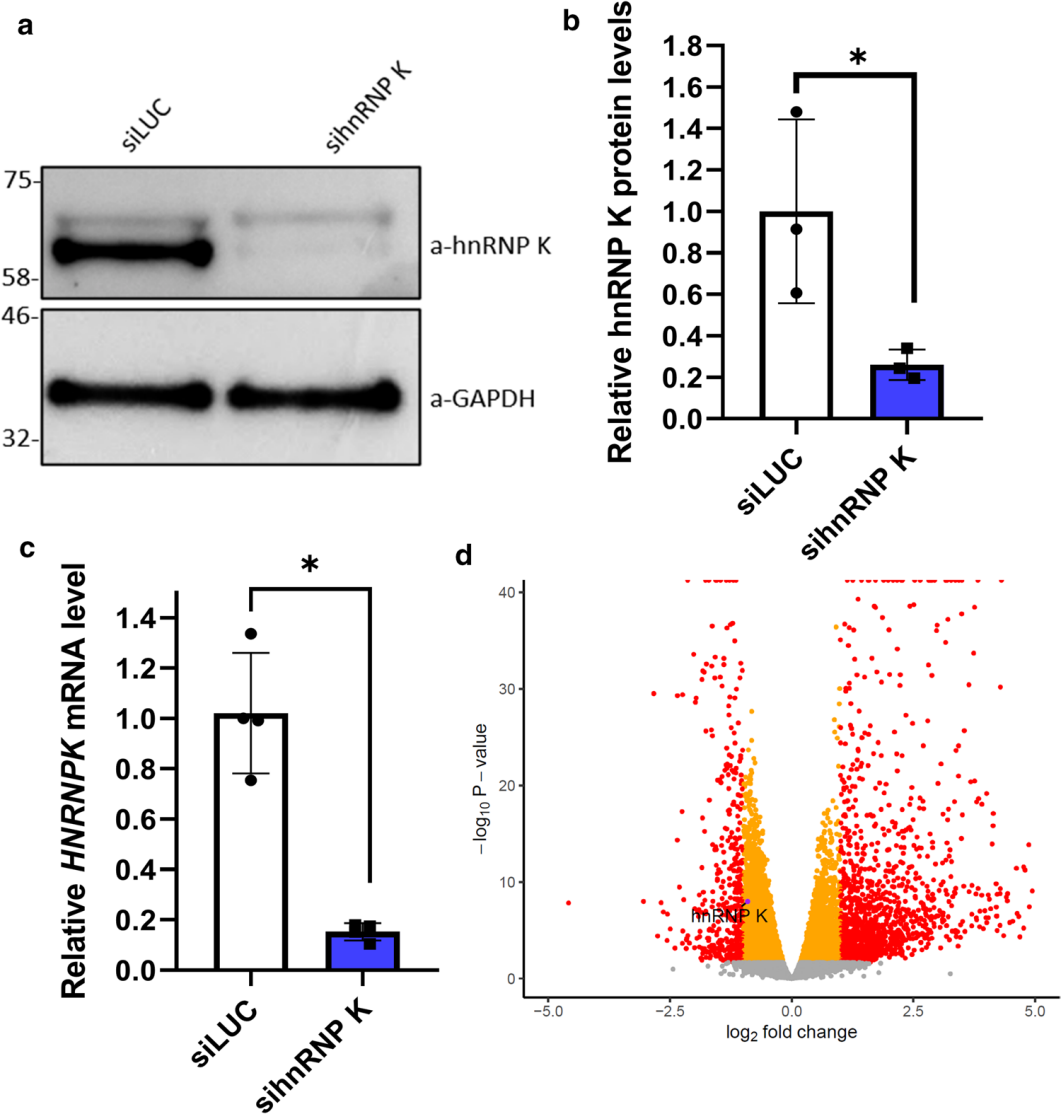
siRNA knockdown of hnRNP K in SH-SY5Y cells leads to widespread gene expression changes. **a** siRNA knockdown of hnRNP K leads to a reduction in protein levels of hnRNP K when compared to siLUC (control) in SH-SY5Y cells. **b** Quantification of western blot analysis of hnRNP K protein levels relative to GAPDH. Protein levels of hnRNP K are reduced to 25% in sihnRNP K SH-SY5Y cells compared to control (*n* = 3, *p* = 0.046, unpaired *t* test). **c** siRNA knockdown of hnRNP K leads to a reduction in mRNA levels of hnRNP k when compared to siLUC (control) in SH-SY5Y cells. (*n* = 4, *p* = 0.029, Mann–Whitney test). **d** Differential gene expression of hnRNP K knockdown finds 1284 genes upregulated and 428 downregulated (FDR < 0.05, |log2 fold change|> 1, genes coloured red). HnRNP K is moderately downregulated (FDR < 0.05, log2 fold change = 0.91, equivalent to 53% reduction, orange points). Genes with *p* < 1e-40 are plotted at the edge of axis

HnRNP K plays a major role in RNA processing in the nucleus. We hypothesised that loss of nuclear hnRNP K leads to aberrant processing of RNA targets, including cryptic exon events. To study this, we knocked down hnRNPK using an siRNA in SH-SY5Y neuroblastoma cells, and performed RNA sequencing (three replicates of each condition). Knockdown was confirmed by western blot (Fig. 4a, b) and RT-PCR (Fig. 4c). Differential expression revealed widespread changes in the transcriptome caused by hnRNP K knockdown, with 1284 genes upregulated and 428 downregulated (|log_2_ fold change|> 1, FDR < 0.05) (Fig. 4d).

**Fig. 5:**
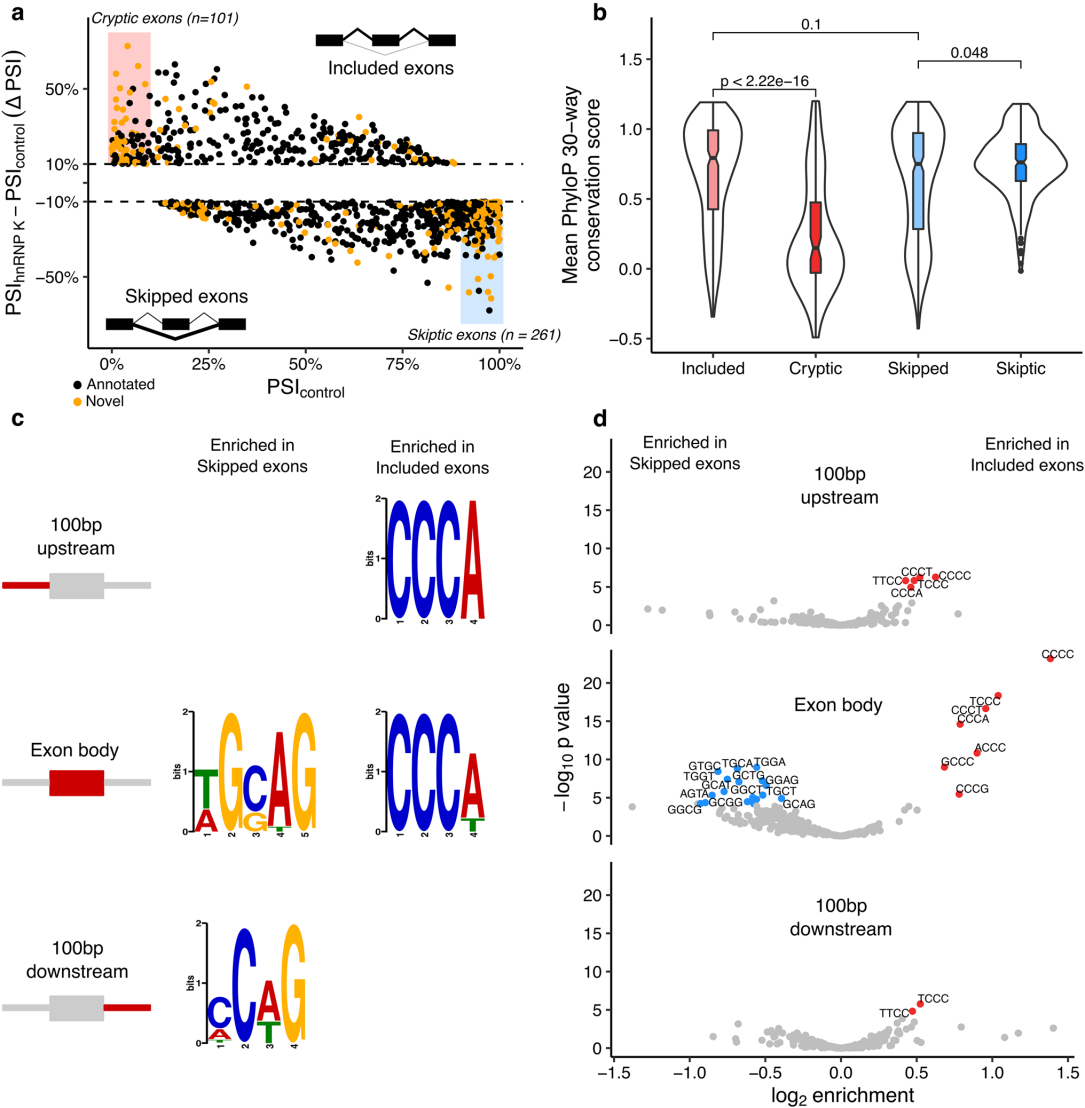
HnRNP K depletion leads to widespread novel splicing. **a** All cassette exons identified with absolute relative inclusion (percentage spliced in, deltaPSI) > 10%. Included exons have a positive deltaPSI and skipped exons have a negative deltaPSI. Exons lowly included in controls (PSI_control_ < 10%) classified as cryptic exons (shaded red). Exons highly included in controls (PSI_control_ > 90%) classified as skiptic exons (shaded blue). **b** Mean phyloP mammalian conservation scores for each class of exon. *p* values are from pairwise nonparametric Wilcoxon tests. **c** The top motifs identified by MEME when comparing the nucleotide sequences of all skipped exons (left column) and all included exons (right column) to the opposing set, split into the 100 bp upstream, the exon body and 100 bp downstream nucleotides. **d** Enrichments of 4-mers between the same sequence sets. *p* values are from a Chi-squared test of equal proportions

Differential splicing analysis found 1039 cassette exons with altered splicing in both directions (Fig. 5a) (|ΔPSI|> 10%, FDR < 0.05), suggesting that hnRNPK has a widespread role in maintaining both gene expression and splicing throughout the neuronal transcriptome. Despite discovering comparable numbers of genes and splicing events, we focussed our attention on splicing. This was due to both the previous evidence of hnRNP K directly binding mRNA and modulating splicing [65], and the possibility of observing hnRNP K-specific splicing events in human post-mortem brains.

Using GENCODE (v30) to annotate the introns used in each cassette exon splicing event, we discovered a large number of hNRNP K-associated cassette exons contained either novel inclusion or novel skipping junctions. Following our previous work on identifying cryptic exons associated with TDP-43 [21, 30, 57], we defined cryptic exons as cassette exons that were lowly included in control samples (PSI_control_ < 10%) with a strong increase in inclusion upon hnRNP K knockdown (ΔPSI > 10%). We identified 101 exons that met our criteria, of which 49 were novel. The inverse of cryptic exons are skiptic exons, where normally constitutively included exons (PSI_control_ > 90%) are skipped in response to perturbation of splicing factors (ΔPSI < − 10%) [21]. We identified 261 skipped exons which met these criteria, 118 of which were novel skipping events.

The presence of cryptic or skiptic exons can lead to degradation of the host transcript by nonsense-mediated decay through frame-shifting or the introduction of a premature stop codon [30]. We compared the direction of differential gene expression with the genes found to harbour cryptic and skiptic exons, as well as other differentially included and skipped exons. Non-canonically spliced genes had a bias towards downregulation, with 34 of the 46 differentially expressed cryptic exon containing genes (74%) downregulated, compared to 58% of a null set of genes matched for similar expression levels (*p* = 9.7e-6; Chi-squared test of equal proportion; Supplementary Fig. 4a, Online resource 2). However, only a small number of cryptic or skiptic exon-containing genes were strongly differentially expressed (|log2 fold change|> 1; Supplementary Fig. 4b). Cryptic exons, and novel exons in general, were less likely to be divisible by three compared to non-cryptic included and annotated exons, suggesting that many could cause frameshifting and protein degradation by nonsense-mediated decay (Supplementary Fig. 4c, d, Online resource 2).

The cryptic exons repressed by TDP-43 are largely non-conserved between species [30, 40], whereas the constitutive exons affected by skiptic splicing tend to be highly conserved [21]. We calculated mean PhyloP 30-way mammalian evolutionary conservation scores for each exon sequence (Fig. 5b). Cryptic exons were less conserved compared with the other included cassette exons (*p* < 1e-16, Wilcoxon test), whereas skiptic exons were slightly more conserved than the rest of the skipped exons (*p* = 0.048). Splitting each group by annotation showed the reduced conservation of cryptic exons was largely due to novel (non-annotated in GENCODE) exons (Supplementary Fig. 4c, Online resource 2).

We next investigated the sequence specificity of hnRNP K-associated splicing events. hnRNP K is known to have a binding preference for cytosine-rich sequences [46, 55, 60]. We took the genomic sequences for the exons strongly included and strongly skipped (|deltaPSI|> 10%) by hnRNP K knockdown and performed differential motif enrichment on the exon sequences, the upstream 100 nucleotides and the downstream 100 nucleotides flanking either side (Fig. 5c). The C-rich motif *CCC[AT] (*square brackets refer to the presence of either nucleotide) was enriched in both the exon bodies and the 5′ flanking sequences of included exons. We observed the *CCC[AT]* sequence within 77% of included exons but only 50% of skipped exons (OR = 1.55; *P* = 1e-17, Chi-squared). When restricting the analysis to only cryptic and skiptic exons this rises to 86% against 48% (OR = 1.8; *P* = 7.7e-11; Supplementary Fig. 3d, Online resource 2). Comparing the skipped exons to the included exons we identified the motif *[TA]G[GC]AG* present in 50% of included exons, but 69% of skipped exons (OR = 0.73; *P* = 2.9e-9; Chi-squared). *[AC]C[AT]G* was also identified in the 3′ flanking sequences of the skipped exons. We verified these motifs by extracting all possible 4-mer nucleotides in the skipped and included exon sets and comparing their frequencies (Fig. 5d, Supplementary Table 6, Online resource 1). C-rich 4-mers were enriched in the exon body, upstream 5′ and downstream 3′ flanking sequences of the included exons when compared to the skipped exons. Conversely, we observed a cluster of TG- and CG-rich sequences enriched in the skipped exon bodies sequences, with no 4-mers enriched in the flanking nucleotides of the skipped exons. This suggests that the exons differentially included under hnRNP K knockdown are directly bound by hnRNP K, whereas the skipped and skiptic exons may be regulated indirectly through other splicing factors.

### HnRNP K mislocalisation in FTLD human brain is associated with the inclusion of cryptic exons

**Fig. 6:**
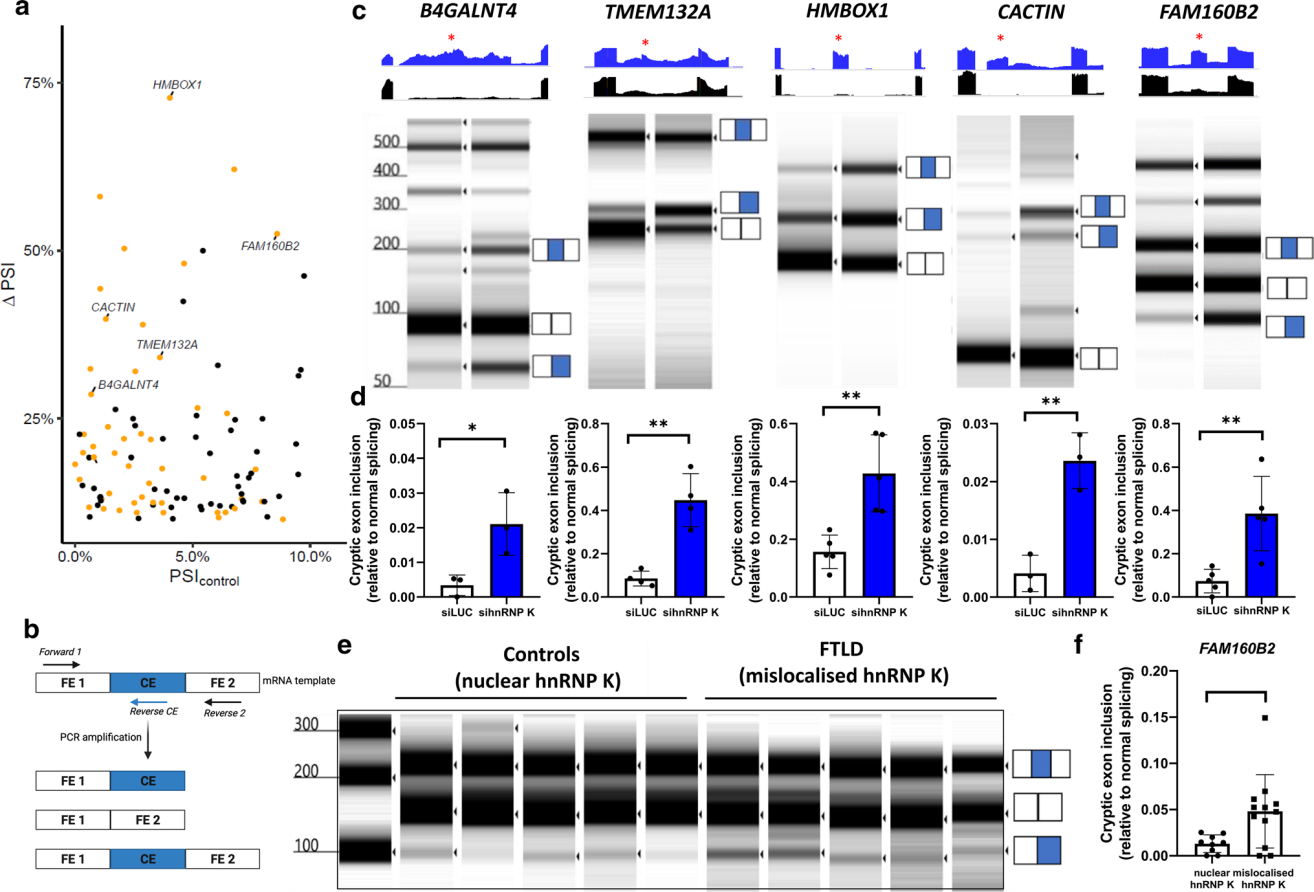
HnRNP K-regulated cryptic exons can be identified in FTLD patient brains. **a** PSI_control_ against deltaPSI for the 101 cryptic exons identified in SH-SY5Y cells. Novel exons are coloured orange. **b** Schematic diagram representing a 3 primer PCR method. A forward and reverse primer on the flanking exons (FE) as well as a reverse primer on the cryptic exon (CE) were used to generate the 3 PCR products shown. **c** Five novel cryptic events were validated in SH-SY5Y cells as a result of sihnRNP K knockdown. The red asterisk (*) in each IGV (integrated genome viewer) trace indicates the cryptic exon. The top trace (blue) corresponds to the sihnRNP K reads, whilst the bottom trace (black) corresponds to the control (siLUC). The presence of the cryptic exon was demonstrated using a 3 primer PCR utilizing primers that flank the cryptic exon (blue), as well as a primer spanning the cryptic exon. **d** Quantification of inclusion of the cryptic exon relative to normal splicing for each of the five events. All five events were more present in sihnRNP K (blue bar) cells compared to siLUC (control, white bar) cells. (*B4GALNT4* (*p* = 0.033), *TMEM132A* (*p* = 0.013), *HMBOX1* (*p* = 0.003)*, CACTIN1* (*p* = 0.004)*,* and *FAM160B2* (*p* = 0.008), unpaired *t* test). **e** Representative image of products from a 3 primer PCR showing an increase in the inclusion of the cryptic exon within *FAM160B2* in FTLD cases with mislocalised hnRNP K. **f** Quantification of inclusion of the cryptic exon relative to normal splicing in *FAM160B2* in cases exhibiting nuclear hnRNP K and cases exhibiting mislocalised hnRNP K (*n* = 9 nuclear hnRNP K, *n* = 12 mislocalised hnRNP K, Mann–Whitney test, *p = 0.019)

HnRNP K knockdown led to the inclusion of 49 cryptic exons which were identified as novel. We proceeded to validate five strong cryptic exon events in RNA targets for *HMBOX1, TMEM132A, CACTIN1, B4GALNT4 and FAM160B2* (Fig. 6a)*.* All five of these events had a deltaPSI > 25% in SH-SY5Y hnRNP K knockdown and are strongly expressed in human brain*.* A three-primer PCR protocol was designed to generate amplicons containing the cryptic exon. Cryptic exons within these five targets were significantly upregulated in hnRNP K knockdown (sihnRNP K) cells compared to control (siLUC) SH-SY5Y cells (*HMBOX1* (*p* = 0.003)*, TMEM132A* (*p* = 0.013)*, CACTIN1* (*p* = 0.004)*, B4GALNT4* (*p* = 0.033) *and FAM160B2* (*p* = 0.008), unpaired *t* test). (Fig. 6b, c). Therefore, hnRNP K knockdown leads to widespread cryptic exon incorporation. We proceeded to investigate whether hnRNP K mislocalisation observed in the frontal cortex of FTLD brains was associated with an increased inclusion of cryptic exons in hnRNP K targets. We found that inclusion of a cryptic exon in *FAM160B2* was significantly upregulated in cases exhibiting widespread hnRNP K mislocalisation (*n* = 12, mean of 44% of sampled images containing at least one mislocalised neuron) compared to cases with predominantly nuclear hnRNP K (*n* = 9, mean of 2% of sampled images with at least one mislocalised neuron) (Mann–Whitney test, *p* = 0.019) (Fig. 6d, e). This suggests that loss of nuclear hnRNP K in the frontal cortex is associated with the inclusion of cryptic exons in targets of hnRNP K.

## Discussion

We have identified a novel pathological event in neurodegenerative disease characterised by the mislocalisation of hnRNP K from the nucleus to the cytoplasm within pyramidal neurons of the frontal cortex in a highly punctate manner. HnRNP K mislocalisation was more frequent in FTLD-TDP A and FTLD-Tau patient brains than in age-matched controls. Mislocalisation was also observed in some elderly controls and indeed was found to correlate with age at death. Hence, it is tempting to speculate its higher occurrence in FTLD may reflect an advanced-ageing phenotype.

Mislocalisation of hnRNP K occurs in neurons that are distinct from those which harbour proteinaceous TDP-43 and Tau inclusions that pathologically define FTLD subtypes. This is novel compared to previous work which has identified several other hnRNPs to be present within TDP-43 and FUS inclusions [15, 24]. There is also an abundance of research on hnRNPs co-depositing with C9orf72-associated pathologies including RNA foci and dipeptide repeat proteins [13, 14, 25, 59]. Interestingly, cytoplasmic hnRNP K puncta within neurons were found to be p62/ubiquitin negative suggesting they are not typical of classical pathological inclusions. We found no colocalisation between hnRNP K puncta and markers of mitochondria, autophagosomes and stress granules. Further investigations, including additional double immunofluorescence, co-immunoprecipitation and biochemical fractionation studies, will be required to characterise the cytoplasmic hnRNP K puncta we observe in pyramidal cells.

We have also identified hnRNP K to have a key physiological role in repressing cryptic splicing in neurons. Using our cryptic exon discovery pipeline, we have identified a large (*n* = 101) number of cryptic exons which are significantly upregulated within our hnRNP K knockdown (SH-SY5Y) cell model at the RNA-seq level, five of which we validated in RNA by PCR. These cryptic exons resemble those found in TDP-43 knockdown experiments in that they are poorly conserved across evolution and likely contribute to reducing the expression of their host genes. Using motif analysis we have demonstrated that the widespread exon inclusion events are likely due to a loss of direct hnRNP K binding to C-rich RNA elements.

We have observed hnRNP K to be frequently and severely depleted from the nucleus of pyramidal neurons in many of our analysed FTLD brains and so hypothesised there to be an elevated degree of cryptic splicing within these cases. Indeed we validated one such event in *FAM160B2* in RNA derived from bulk frontal cortex homogenate. Validation of other cryptic events in addition to *FAM160B2* have been tried but without the success we have obtained for this specific splicing change. However, it must be pointed out that these analyses were performed in “bulk” tissue, and therefore, the sensitivity of the approach was distant from ideal. In the future, other types of approaches such as FISH or CISH techniques will be used to obtain a better appraisal of cryptic exon inclusion in the presence of hnRNP K nuclear depletion. Nonetheless, the specific detection of the *FAM160B2* in patient samples that exhibit hnRNP K mislocalisation served as a proof-of-principle that loss of nuclear hnRNP K in human brain can result in splicing changes. Hence, like TDP-43 and indeed several other RBPs, hnRNP K appears to have a crucial homeostatic role in maintaining transcriptomic stability that may be compromised in FTLD.

Future work utilising single-cell approaches and alternative neuronal knockdown models of hnRNP K will be important to identify cryptic events that are mechanistically relevant to the neurodegeneration phenotype. Events that lead to functional depletion of proteins that enhance neuronal vulnerability (analogous to truncated stathmin-2 in TDP-43 depletion models) or even the elevation of novel protein isoforms that evade nonsense-mediated decay altogether will be of special interest [48, 57]. Indeed, investigating the potential interaction between hnRNP K and TDP-43 within FTLD-related RNA misprocessing may be of mechanistic interest in itself. The two RBPs have been found to colocalise within stress granules (SGs), with TDP-43 accumulation into SGs being dependent on prior phosphorylation of hnRNP K by cyclin-dependent kinase 2 [52]. Both proteins were also found to be robustly nuclear depleted within iPSC-derived motor neurons subjected to osmotic stress [26]. HnRNP K has also been identified as a key driver for nuclear retention of long non-coding RNA (lncRNA) Malat1 which, when disrupted, leads to increased Malat1-TDP-43 binding and an increased propensity for TDP-43 aggregation as has been observed in ALS patients [54, 62]. Therefore, protein levels of hnRNP K as well as its subcellular localisation and phosphorylation status may be mechanistically crucial in maintaining normal stress granule assembly/dissolution and TDP-43 proteostasis [52, 54]. In support of an interplay between these proteins, hnRNP K expression was found to be impaired in several TDP-43 mutant cell and murine models and was also found to be a modifier of TDP-43 pathology in Drosophila and cell-based models of TDP-43 overexpression/depletion [1, 51]. Potentially overlapping cryptic hits could illuminate converging pathways and networks of dysregulation in TDP-43 proteinopathies.

Further lines of enquiry may include those aimed at understanding the specific vulnerability of pyramidal neurons. Laser capture microdissection and single-cell RNA-seq paradigms will prove powerful in delineating transcriptomic differences between cortical neuronal cell types. As is the case with several other hnRNPs, it will also be important to determine whether hnRNP K autoregulates its own expression and whether or not this mechanism is dysregulated in neurons with mislocalised protein. Establishing whether any sequence mutations or post-translational modifications of hnRNP K are more or less associated with mislocalisation will also be insightful in this regard.

To conclude, our discovery of hnRNP K mislocalisation in FTLD and induced cryptic exon inclusion following hnRNP K nuclear depletion adds to the growing body of research of RNA misprocessing in FTLD and ALS pathogenesis. HnRNP K joins other hnRNP proteins that have been found to be pathologically dysregulated in these disorders including TDP-43, FUS and hnRNP A1. It will also be important to establish whether hnRNP K mislocalisation and its impact on RNA splicing is relevant to other neurodegenerative diseases. A greater understanding of the pathomechanisic underpinnings and consequences of hnRNP K mislocalisation in neurodegeneration may have the potential to identify novel biomarkers and therapeutic targets.

## Data and code availability

Raw RNA sequencing data, as well as processed gene expression and splice junction counts, have been uploaded to the Gene Expression Omnibus (GEO) with accession GSE171090. All code used to generate figures is hosted on github: https://github.com/jackhump/hnRNPK/. Script for classifying cassette exons from Leafcutter results: https://github.com/davidaknowles/leafcutter/blob/classify_clusters/leafviz/classify_clusters.R.

## Supplementary Information

Below is the link to the electronic supplementary material.

Supplementary file1 Supplementary Table1 Antibodies used for immunohistochemistry. Supplementary Table2 Full differential gene expression results for hnRPNK siRNA knockdown. Supplementary Table 3 Differential splicing results for all cassette exons for the hnRNPK siRNA knockdown. Supplementary Table 4 Motif enrichment analysis for all 2mers. Supplementary Table 5 Motif enrichment analysis for all 3mers. Supplementary Table 6 Motif enrichment analysis for all 4mers. Supplementary Table 7 Sequences for all primers used for validation (XLSX 2292 KB)

Supplementary file2 Supplementary Fig. 1 Examples of hnRNP K mislocalisation in frontal cortex pyramidal neurons. Examples from three separate (a) FTLD-TDP A, (b) FTLD-TDP C and (c) FTLD-Tau cases. (d) Examples of hnRNP K-stained pyramidal neurons in age-matched controls (first 2 panels, age at death = 67 and 68, respectively) and hnRNP K mislocalisation in pyramidal neurons of an elderly control (age at death = 86, final panel). (e) ALS (n = 7), FTLD-TDP A (n = 28), FTLD-TDP C (n = 12), FTLD-Tau (n = 5) and control (n = 18) cohorts were age-matched with no significant difference between mean age at death. (f) FTLD-TDP A and FTLD-Tau cases exhibited a significantly higher proportion of neurons (%) with hnRNP K mislocalisation compared to age-matched controls. Supplementary Fig. 2 Neurons that exhibit hnRNP K mislocalisation are independent of those that exhibit Tau-inclusions. Representative images of double-label immunofluorescence in pyramidal neurons with normal (a) and abnormal (b) hnRNP K localisation in FTLD-Tau frontal cortex with phospho-tau (AT8) marker demonstrating no clear colocalisation of cytoplasmic puncta. Orange arrows point to AT8-positive inclusions and scale bars are as indicated in the first row. Supplementary Fig. 3 Mislocalised cytoplasmic hnRNP K does not colocalise with mitochondria, autophagy or stress granule markers. (a and b) Representative images of double-label immunofluorescence in pyramidal neurons with normal (a) and abnormal (b) hnRNP K localisation in control and FTLD-TDP A frontal cortex, respectively, with mitochondrial marker VDAC-1. c, d show the spatial relationship between normal (c) and abnormally (d) localised hnRNP K with autophagy marker LC3 and (e and f) shows the same cases again with stress granule/RNA-binding protein marker G3PB2. In all cases no clear colocalisation was observed within cytoplasmic hnRNP K puncta. Supplementary Fig. 4 HnRNP K knockdown leads to widespread novel splicing and differential expression. (a) Differential expression directions for the sets of genes containing each type of exon. Percentages refer to the percentage of each set that have a log2 fold change < 0 and FDR < 0.05. “Weak splicing”refers to exons with |deltaPSI| < 10%. No splicing refers to a set of genes without differential splicing, matched for expression levels. (b) Differential gene expression fold changes of genes containing skiptic (blue) or cryptic (red) exons. Genes with |log2 fold change| > 1 are labelled. (c, d) Frame-preserving abilities of the hnNRNP K associated exons. Frame preservation defined as an exon whose width is divisible by 3. The null expectation (dotted line) would be 1/3 of a set of exons preserving reading frame. *p* values refer to the Chi-squared test of the proportion of each set compared to the null. (c) Exons split by functional prediction. (d) Exons split by annotation status. (e) PhyloP 30-way conservation scores for each exon, split by exon type and whether the inclusion or skipping junctions are annotated in GENCODE v30. Novel exons of all types have substantially lower conservation. (f) 4-mer enrichment analysis as in Figure 5d, restricted to only cryptic and skiptic exons. (PDF 959 KB)
