# Supplementary material for: HnRNP K mislocalisation is a novel protein pathology of frontotemporal lobar degeneration and ageing and leads to cryptic splicing
Source: Acta Neuropathol. 2021 Jul 18;142(4):609–27. doi: 10.1007/s00401-021-02340-0 (PMC8423707; doi:10.1007/s00401-021-02340-0)
Supplement: Supplementary file 2 — Supplementary file2 Supplementary Fig. 1 Examples of hnRNP K mislocalisation in frontal cortex pyramidal neurons. Examples from three separate (a) FTLD-TDP A, (b) FTLD-TDP C and (c) FTLD-Tau cases. (d) Examples of hnRNP K-stained pyramidal neurons in age-matched controls (first 2 panels, age at death = 67 and 68, respectively) and hnRNP K mislocalisation in pyramidal neurons of an elderly control (age at death = 86, final panel). (e) ALS (n = 7), FTLD-TDP A (n = 28), FTLD-TDP C (n = 12), FTLD-Tau (n = 5) and control (n = 18) cohorts were age-matched with no significant difference between mean age at death. (f) FTLD-TDP A and FTLD-Tau cases exhibited a significantly higher proportion of neurons (%) with hnRNP K mislocalisation compared to age-matched controls. Supplementary Fig. 2 Neurons that exhibit hnRNP K mislocalisation are independent of those that exhibit Tau-inclusions. Representative images of double-label immunofluorescence in pyramidal neurons with normal (a) and abnormal (b) hnRNP K localisation in FTLD-Tau frontal cortex with phospho-tau (AT8) marker demonstrating no clear colocalisation of cytoplasmic puncta. Orange arrows point to AT8-positive inclusions and scale bars are as indicated in the first row. Supplementary Fig. 3 Mislocalised cytoplasmic hnRNP K does not colocalise with mitochondria, autophagy or stress granule markers. (a and b) Representative images of double-label immunofluorescence in pyramidal neurons with normal (a) and abnormal (b) hnRNP K localisation in control and FTLD-TDP A frontal cortex, respectively, with mitochondrial marker VDAC-1. c, d show the spatial relationship between normal (c) and abnormally (d) localised hnRNP K with autophagy marker LC3 and (e and f) shows the same cases again with stress granule/RNA-binding protein marker G3PB2. In all cases no clear colocalisation was observed within cytoplasmic hnRNP K puncta. Supplementary Fig. 4 HnRNP K knockdown leads to widespread novel splicing and differential expression. ( [file 401_2021_2340_MOESM2_ESM.pdf]

Online resource 1 – Supplementary figures and legends

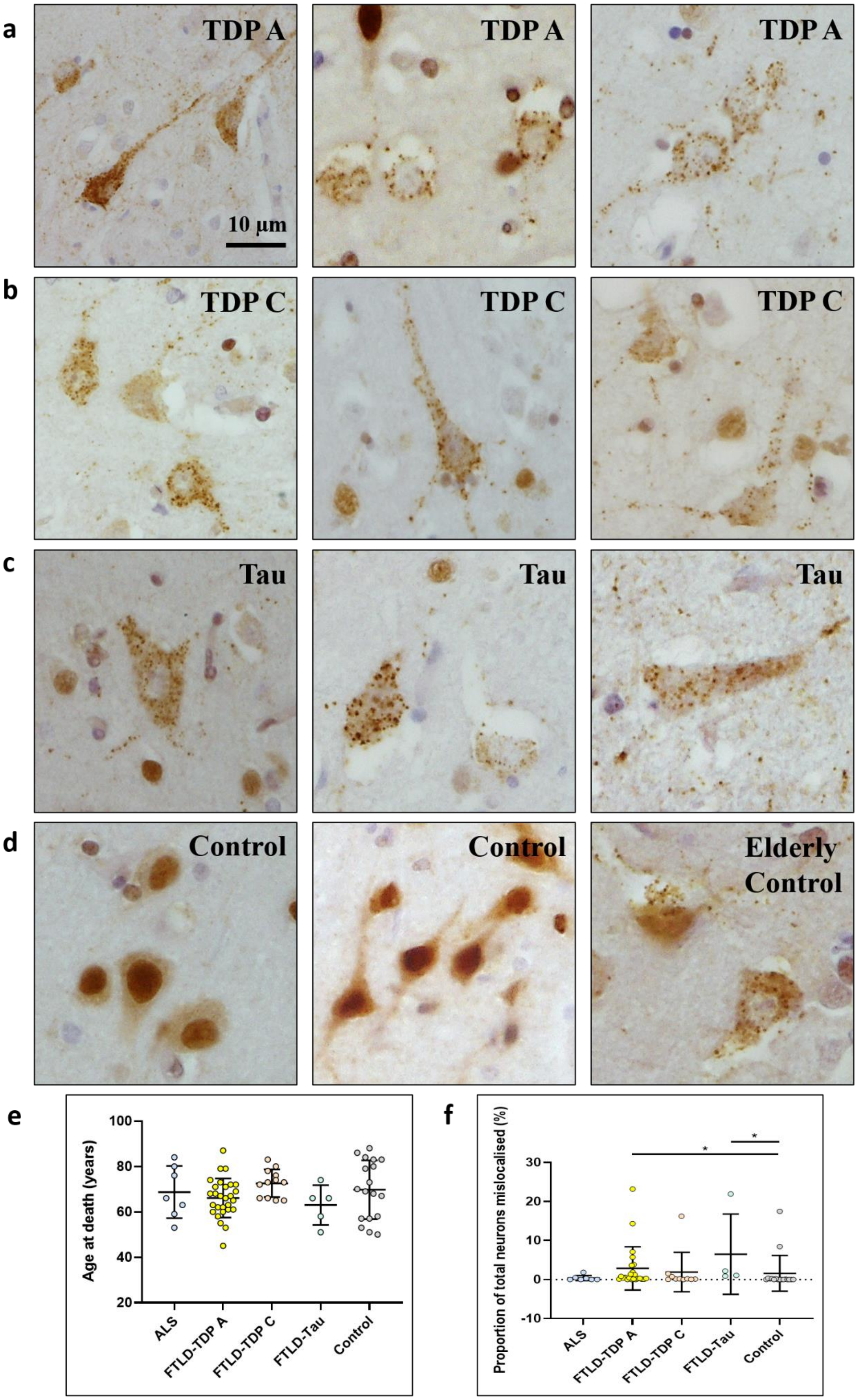

Supplementary Fig. 1

Examples of hnRNP K mislocalisation in frontal cortex pyramidal neurons. Examples from three separate **(a)** FTLD-TDP A, **(b)** FTLD-TDP C and **(c)** FTLD-Tau cases. **(d)** Examples of hnRNP K-stained pyramidal neurons in age-matched controls (first 2 panels, age at death = 67 and 68 respectively) and hnRNP K mislocalisation in pyramidal neurons of an elderly control (age at death = 86, final panel). **(e)** ALS (n = 7), FTLD-TDP A (n = 28), FTLD-TDP C (n = 12), FTLD-Tau (n = 5) and control (n = 18) cohorts were age-matched with no significant difference between mean age at death. **(f)** FTLD-TDP A and FTLD-Tau cases exhibited a significantly higher proportion of neurons (%) with hnRNP K mislocalisation compared to age-matched controls.

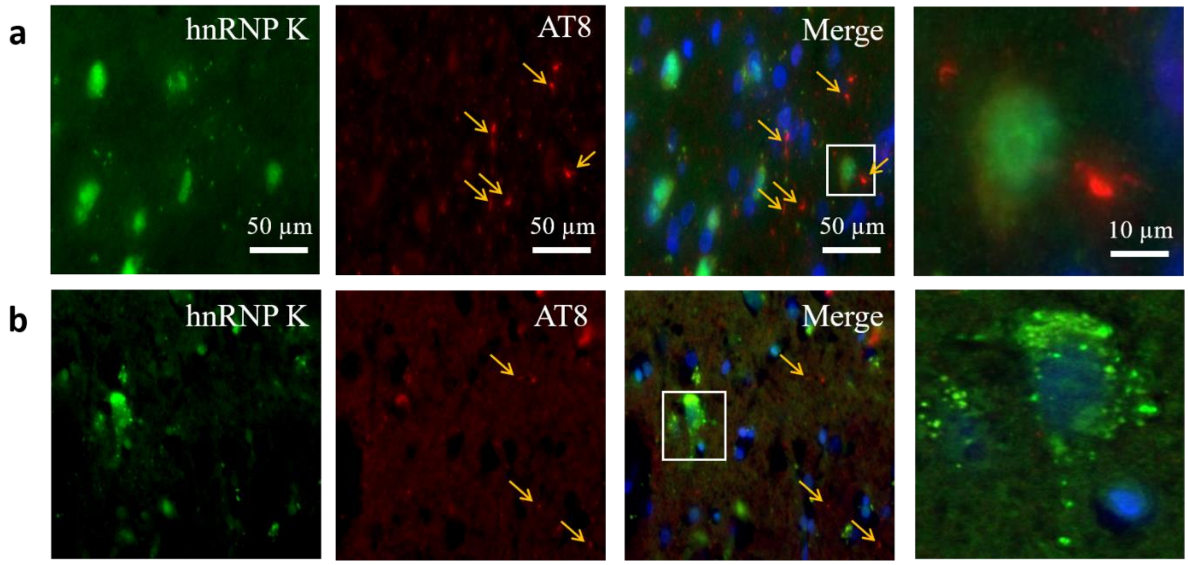

Supplementary Fig. 2

Neurons that exhibit hnRNP K mislocalisation are independent of those that exhibit Tau-inclusions. Representative images of double-label immunofluorescence in pyramidal neurons with normal **(a)** and abnormal **(b)** hnRNP K localisation in FTLD-Tau frontal cortex with phospho-tau (AT8) marker demonstrating no clear colocalisation of cytoplasmic puncta. Orange arrows point to AT8-positive inclusions and scale bars are as indicated in the first row.

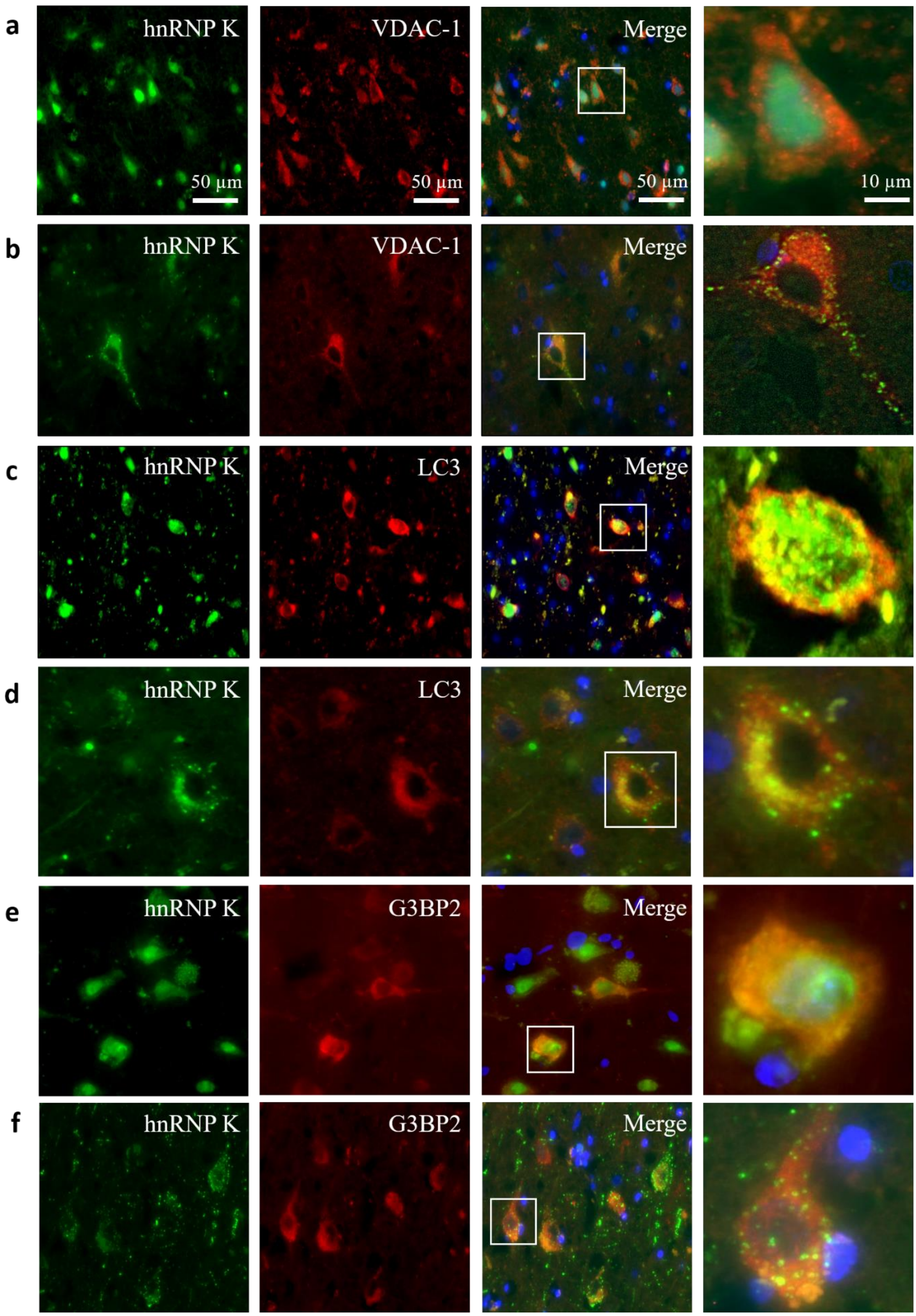
a
hnRNP K
VDAC-1
Merge
50 µm
50 µm
50 µm
10 µm
b
hnRNP K
VDAC-1
Merge
c
hnRNP K
LC3
Merge
d
hnRNP K
LC3
Merge
e
hnRNP K
G3BP2
Merge
f
hnRNP K
G3BP2
Merge

Supplementary Fig. 3

Mislocalised cytoplasmic hnRNP K does not colocalise with mitochondria, autophagy or stress granule markers. (a and **b)** Representative images of double-label immunofluorescence in pyramidal neurons with normal **(a)** and abnormal **(b)** hnRNP K localisation in control and FTLD-TDP A frontal cortex respectively with mitochondrial marker VDAC-1. **(c** and **d)** shows the spatial relationship between normal **(c)** and abnormally **(d)** localised hnRNP K with autophagy marker LC3 and **(e** and **f)** shows the same cases again with stress granule / RNA-binding protein marker G3PB2. In all cases no clear colocalisation was observed within cytoplasmic hnRNP K puncta.

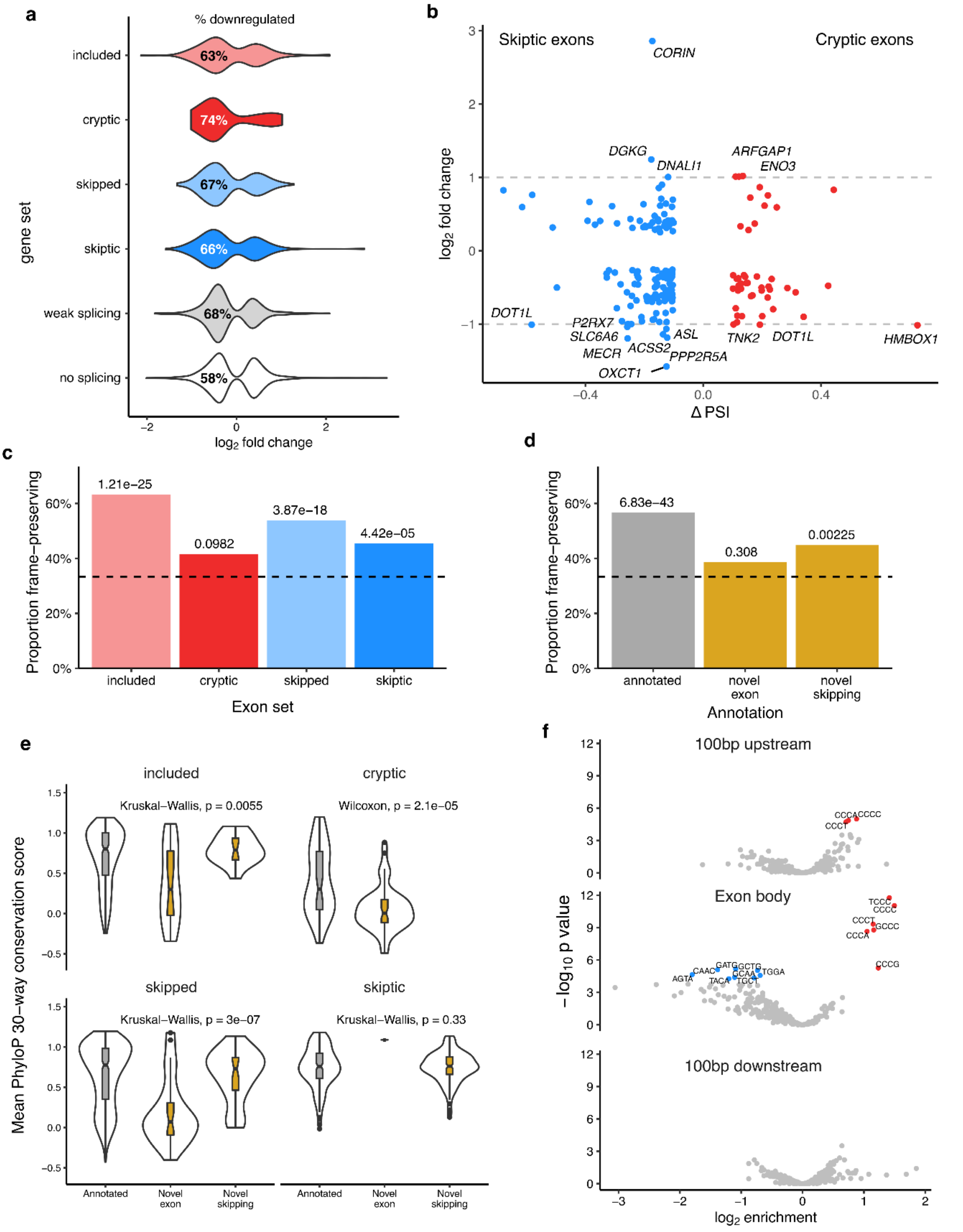

Supplementary Fig. 4

HnRNP K knockdown leads to widespread novel splicing and differential expression. (**a**) Differential expression directions for the sets of genes containing each type of exon. Percentages refer to the percentage of each set that have a $\log_2$ fold change < 0 and FDR < 0.05. “Weak splicing” refers to exons with |deltaPSI| < 10%. No splicing refers to a set of genes without differential splicing, matched for expression levels. (**b**) Differential gene expression fold changes of genes containing skiptic (blue) or cryptic (red) exons. Genes with

|log2 fold change| > 1 are labelled. (**c-d**) Frame-preserving abilities of the hnNRNP K associated exons. Frame preservation defined as an exon whose width is divisible by 3. The null expectation (dotted line) would be ⅓ of a set of exons preserving reading frame. P-values refer to the chi-squared test of the proportion of each set compared to the null. (**c**) Exons split by functional prediction. (**d**) Exons split by annotation status. (**e**) PhyloP 30-way conservation scores for each exon, split by exon type and whether the inclusion or skipping junctions are annotated in GENCODE v30. Novel exons of all types have substantially lower conservation. (**f**) 4-mer enrichment analysis as in Figure 5d, restricted to only cryptic and skiptic exons.
